# Perception as self-organizing interaction: embodied cognition, artificial intelligence, and autism

**DOI:** 10.3389/fpsyg.2026.1803234

**Published:** 2026-03-30

**Authors:** Gerry Leisman, Raymond Roy, Rahela Alfasi

**Affiliations:** 1 Movement and Cognition Laboratory, Department of Physical Therapy, University of Haifa, Haifa, Israel; 2 Resonance Therapeutics Laboratory, Department of Neurology, University of the Medical Sciences of Havana, Havana, Cuba; 3 Department of Neuroscience, Neuroscience of Imagination, Cognition, and Emotion Research Lab, Carleton University, Ottawa, ON, Canada

**Keywords:** autism spectrum disorder (ASD), embodied artificial intelligence, embodied cognition, predictive processing, self-organizing perception, sensorimotor integration

## Abstract

Perception has traditionally been conceptualized as the internal reconstruction of external stimuli, both in cognitive science and in artificial intelligence (AI). In this representational view, sensory systems transform input into internal models that guide cognition and action. However, converging evidence from neuroscience, perceptual science, developmental psychology, autism research, robotics, and contemporary AI increasingly challenges this assumption. Across these domains, perception appears to emerge through active, embodied engagement with the environment rather than through passive signal processing or static internal representation. Embodied cognition theories propose that perceptual meaning arises from lawful relations among bodily constraints, action, temporal coordination, and environmental feedback, emphasizing perception as an ongoing process of interaction. In parallel, recent advances in AI have shifted away from purely feedforward or data-driven perceptual architectures toward closed-loop, predictive, and self-organizing systems in which perception and action are inseparable components of adaptive behavior. Approaches such as embodied reinforcement learning, active inference, and world-model-based learning increasingly treat perception as emerging through sensorimotor interaction and temporally structured regulation rather than inference alone. This theoretical paper integrates embodied cognition with contemporary AI-driven models of perception, arguing that embodiment functions as a generative constraint enabling robust, context-sensitive, and developmentally grounded sensory cognition across biological and artificial systems. We further extend this framework to autism spectrum disorder (ASD), proposing that many sensory–perceptual differences in autism can be understood as variations in embodied self-organization, predictive regulation, and temporal coordination rather than as deficits in abstract cognition. Finally, we discuss how embodied AI systems can serve as formal testbeds for exploring autism-relevant perceptual mechanisms and for designing adaptive, interaction-based technologies that support perceptual coherence without imposing normative behavioral models.

## 1 Introduction

Perception occupies a foundational position in theories of cognition, shaping how organisms and artificial agents detect structure in the environment, generate expectations, and coordinate action over time. Classical approaches in cognitive science conceptualized perception as the transformation of sensory input into internal representations that could be manipulated by cognitive systems to support reasoning and decision-making. Within this framework, perception was treated as an inferential pipeline proceeding from sensation to representation and then to action, with each stage operating according to separable computational principles. Although historically influential, this view has increasingly been questioned by empirical and theoretical work demonstrating that perceptual processing is deeply intertwined with action, context, and bodily state rather than functioning as an isolated input module (Clark, 2013; Engel et al., 2013).

Early artificial intelligence systems largely inherited this representational architecture, modeling perception as feature extraction followed by symbolic or statistical inference (Brooks, 1991). While such systems achieved notable successes in constrained domains, their limitations became apparent in complex, dynamic environments requiring adaptation, generalization, and real-time interaction. Contemporary analyses have shown that disembodied AI systems often remain brittle outside their training distributions and struggle to cope with contextual variation, highlighting the inadequacy of perception models that lack sensorimotor grounding (LeCun et al., 2015; Lake et al., 2017, 2023; Marcus, 2018). These limitations parallel long-standing critiques within perceptual science, where purely stimulus-driven models have failed to account for context sensitivity and action dependence in human perception.

Over the past two decades, converging evidence from neuroscience and perceptual psychology has increasingly challenged the notion that perception is a passive decoding of sensory input. Neurophysiological studies demonstrate extensive bidirectional coupling between sensory and motor systems, with action-related signals modulating early sensory processing and shaping perceptual expectations (Rizzolatti and Sinigaglia, 2016; Summerfield and de Lange, 2014). Behavioral research further shows that perceived object properties, distances, and affordances systematically depend on an observer’s action capabilities and bodily constraints, indicating that perception is scaled to possibilities for action rather than reflecting objective stimulus properties alone (Witt, 2011; Proffitt, 2006). Together, these findings support a view of perception as an active, context-sensitive process embedded in ongoing interaction with the environment.

Embodied cognition offers a theoretical framework capable of integrating these empirical observations by treating perception as an emergent property of sensorimotor engagement rather than as the construction of internal representations detached from action. From this perspective, perceptual meaning arises through lawful relations between movement, bodily constraints, and environmental feedback, emphasizing interaction over internal modeling (Wilson, 2002; Varela et al., 1991). Importantly, embodied approaches do not deny the existence of internal neural processes but reject the assumption that perception can be adequately explained without reference to the body and its actions. Recent syntheses in perceptual science increasingly converge on this position, highlighting the constitutive role of action, posture, and temporal coordination in shaping perceptual experience (Engel et al., 2013; Clark, 2013).

A parallel shift has occurred in artificial intelligence research. Advances in robotics, embodied reinforcement learning, active inference, and world-model-based learning increasingly treat perception and action as inseparable components of adaptive behavior. Rather than relying on static input–output mappings, embodied AI systems learn through closed-loop interaction, using action to structure sensory input and reduce uncertainty over time (Levine et al., 2023; Hafner et al., 2023; Pezzulo et al., 2024). These systems demonstrate that embodiment and temporal dynamics can function as computational resources, enabling perceptual organization to emerge through interaction rather than exhaustive internal representation. Such developments underscore that embodiment is not merely an implementation detail but a generative constraint shaping perceptual learning and robustness.

The present paper develops a theoretical synthesis linking embodied cognition with contemporary AI-driven models of perception, arguing that self-organizing, embodied interaction constitutes a necessary condition for robust sensory cognition. We propose that perception is best understood as an action-dependent, predictive, and temporally structured process that emerges through continuous coupling among body, environment, and learning dynamics. Figures presented later in the paper contrast classical representational architectures with embodied, self-organizing models in which perceptual structure arises through dynamic interaction rather than centralized control.

Finally, this framework is extended to autism spectrum disorder, where differences in sensory processing, motor coordination, and perceptual integration are increasingly recognized as core and early-emerging features. Large-scale reviews and developmental studies indicate that atypical sensory responsivity and motor variability often precede social-communicative differences, suggesting that perception in autism may follow distinct developmental trajectories shaped by embodied interaction (Thye et al., 2022; Iverson et al., 2021; West et al., 2022). Rather than conceptualizing these differences as deficits in abstract cognition or representation, the present approach situates autism within a process-oriented account of perception that emphasizes self-organization, predictive regulation, and temporal coordination across development. By integrating perceptual science, autism research, and embodied AI, this paper aims to advance a unified framework capable of accommodating both biological diversity and artificial intelligence within a common explanatory space.

## 2 Embodied cognition as a theory of perception

Embodied cognition reframes perception as a process that emerges through sustained interaction between an agent and its environment, rather than as the internal reconstruction of an external world from sensory input alone. Within this framework, perception is not treated as a preliminary computational stage that precedes cognition and action, but as an ongoing regulatory process through which agents actively structure sensory information by moving, exploring, and engaging with the world. Perceptual organization is thus inseparable from bodily constraints, action capabilities, and the temporal dynamics of interaction. This perspective challenges long-standing assumptions in perceptual science that stable perception can be fully explained by stimulus-driven encoding or internal representational inference.

Over the last two decades, empirical findings across perceptual psychology, neuroscience, and developmental science have increasingly supported this interaction-centered view. Studies of visual, auditory, and multisensory perception demonstrate that perceptual experience is systematically modulated by posture, movement, effort, and task demands, indicating that perception is scaled to an organism’s possibilities for action rather than determined solely by objective stimulus properties (Proffitt, 2006; Witt, 2011). Neurophysiological evidence further shows that sensory processing is shaped by motor intentions and predictive signals, with action-related activity influencing early stages of perceptual processing (Summerfield and de Lange, 2014; Rizzolatti and Sinigaglia, 2016). Together, these findings undermine the notion that perception operates as a passive input channel and instead point toward a model in which perception is enacted through embodied engagement.

From an embodied cognition perspective, the central explanatory task of perceptual science shifts from identifying how internal representations mirror the world to understanding how agents achieve perceptual stability and meaning through action-dependent regulation. Perceptual coherence, on this view, does not require detailed internal models of the environment but emerges through the reliable coordination of sensory input and motor activity over time. This emphasis on regulation, rather than reconstruction, provides a unifying framework for understanding perception across biological and artificial systems, as well as across typical and atypical developmental trajectories.

### 2.1 Perception as enacted, not represented

A core commitment of embodied cognition is that perception is enacted through ongoing interaction rather than encoded as a set of static internal representations. This claim does not deny the existence of neural processes or internal dynamics, but it rejects the assumption that perceptual experience depends primarily on the construction of content-rich internal models detached from action. Instead, perception is understood as arising from an agent’s capacity to engage with the environment in ways that are constrained by its body, its sensorimotor repertoire, and the structure of the world itself (Wilson, 2002; Noë, 2004; Marr, 1982; Pylyshyn, 1984).

Within this enactive framework, perceptual stability is achieved through the reliable coordination of action and sensation rather than through the accumulation of increasingly accurate internal descriptions. For example, visual perception depends on lawful patterns of change generated by eye, head, and body movements, while tactile and auditory perception similarly rely on active sampling of sensory information. Empirical studies show that when normal patterns of movement are disrupted, perceptual experience is altered even when the physical stimulus remains constant, demonstrating that action plays a constitutive role in perception rather than serving merely as an output of perceptual processing (Engel et al., 2013; Clark, 2013).

This approach offers a principled response to classic problems in perceptual theory, including underdetermination and ambiguity. Rather than positing that the brain resolves ambiguous sensory input by inferring the most likely hidden causes, embodied theories emphasize that ambiguity is reduced through active exploration that structures sensory input over time. Perception becomes reliable not because the system internally reconstructs the world, but because it can act in ways that generate predictable sensory consequences. In this sense, perception is better characterized as a form of skilled engagement with the environment than as the recovery of objective stimulus properties.

**Figure 1:**
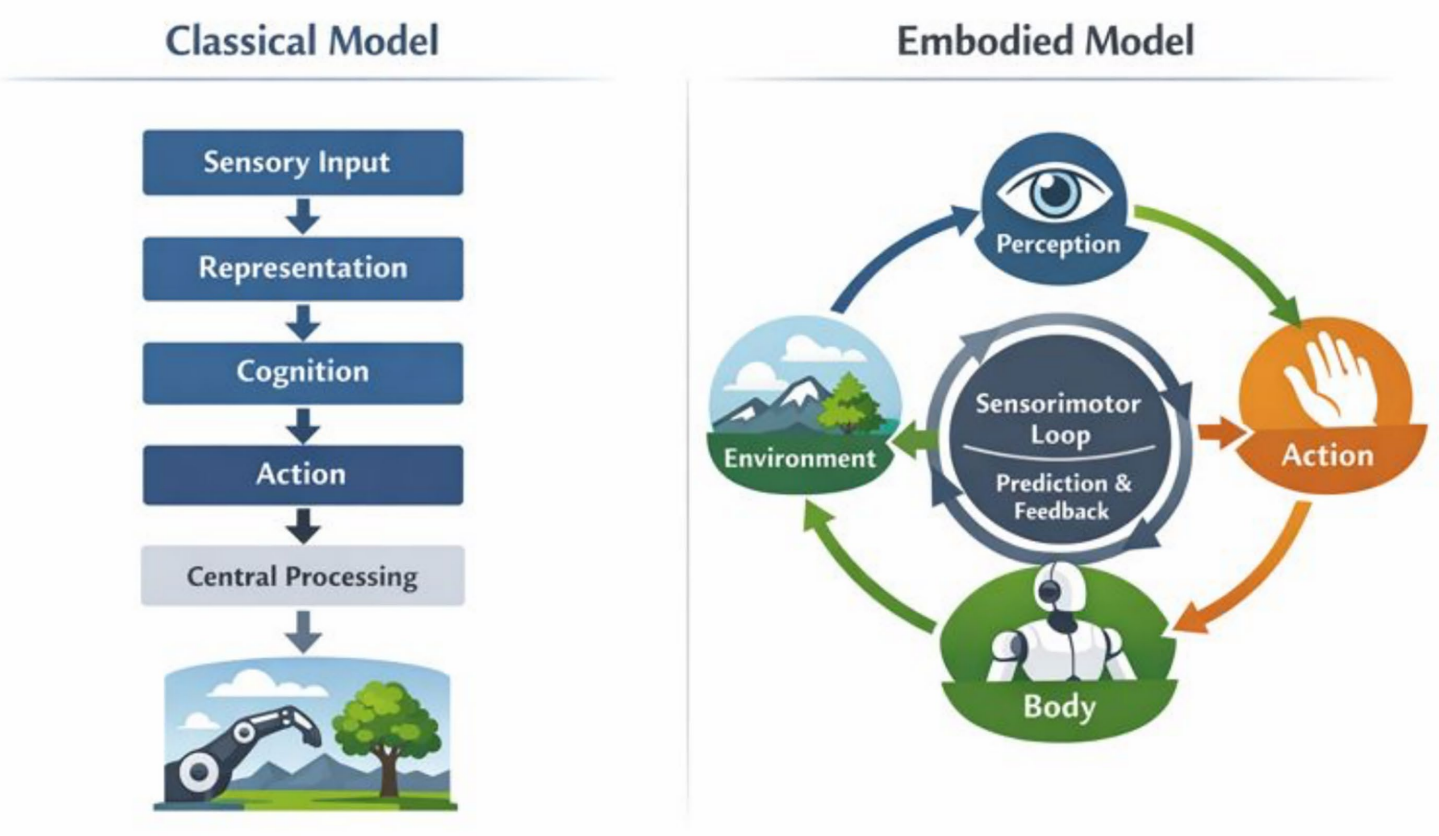
Classical representational versus embodied, self-organizing models of perception. The left panel illustrates a traditional disembodied architecture in which perception is modeled as the linear transformation of sensory input into internal representations that subsequently guide cognition and action. Information flow is predominantly unidirectional, with perception preceding decision-making and motor output. The right panel depicts an embodied, self-organizing framework in which perception emerges through continuous, bidirectional sensorimotor interaction among the body, environment, and predictive processes. In this model, action actively structures sensory input, learning is driven by feedback and prediction error, and perceptual organization arises through dynamic coupling rather than centralized representational control.

Neuroscientific evidence increasingly supports this view by demonstrating that perceptual processing is shaped by anticipatory signals linked to intended and ongoing actions. Motor planning and prediction influence sensory cortices, blurring the distinction between perception and action at both functional and neural levels (Rizzolatti and Sinigaglia, 2016; Summerfield and de Lange, 2014). These findings suggest that perception is inherently prospective and action-oriented, aligning closely with embodied accounts that treat perception as something organisms do rather than something that happens to them. Figure 1 contrasts this classical representational view of perception with an embodied, self-organizing alternative, highlighting the shift from unidirectional, input-driven processing to continuous, bidirectional sensorimotor coupling in which action actively structures perceptual organization.

### 2.2 Sensorimotor contingencies and perceptual meaning

Sensorimotor contingency theory provides a more formal account of how embodied interaction gives rise to perceptual experience by focusing on the lawful relationships between action and sensory change. According to this approach, perceptual qualities are not properties of sensory signals themselves but are defined by the regularities governing how sensory input varies as an agent moves and acts in the world (O’Regan and Noë, 2001). What an agent perceives thus depends on its mastery of these contingencies, rather than on the passive reception of sensory information.

This framework explains why perception remains stable despite variability in sensory input. By learning the sensorimotor dependencies that link action to sensation, organisms can maintain perceptual coherence under changing conditions, such as variations in viewpoint, illumination, or background noise. Experimental manipulations that alter sensorimotor contingencies, including tool use, virtual environments, and altered sensory feedback, reliably produce corresponding changes in perceptual experience, supporting the claim that perceptual meaning is grounded in action-dependent regularities rather than stimulus features alone (Engel et al., 2013; Clark, 2013).

Developmental research provides particularly strong support for this view. Perceptual abilities emerge in tandem with the development of self-generated movement, and active exploration plays a critical role in calibrating sensory systems. Studies of infant development show that restrictions on motor experience delay or alter the emergence of depth perception, object perception, and multisensory integration, indicating that perception is learned through embodied interaction rather than passive exposure to sensory input (Campos et al., 2000; Adolph and Robinson, 2015). These findings suggest that perceptual organization is not pre-specified but gradually self-organizes as agents learn how their actions transform sensory input over time.

Crucially, sensorimotor contingency theory highlights the temporal dimension of perception. Mastery of sensorimotor regularities requires integrating information across time, linking past actions to present sensations and future expectations. Perception, in this sense, is inherently dynamic and anticipatory, depending on the coordination of action and sensation over multiple timescales. This emphasis on temporally extended interaction provides a natural bridge to later sections of the paper, where predictive processing and rhythmic coordination are examined as key mechanisms supporting perceptual stability in both biological and artificial systems.

Taken together, embodied cognition and sensorimotor contingency theory offer a coherent account of perception as an emergent, self-organizing process grounded in action, bodily constraints, and temporal structure. Rather than treating perception as the reconstruction of a pre-given world, these approaches emphasize how perceptual meaning arises through interaction, learning, and regulation. This interaction-centered view provides the conceptual foundation for the subsequent analysis of disembodied models in cognitive science and AI, as well as for the extension of embodied perceptual theory to autism spectrum disorder and AI-driven perceptual systems.

### 2.3 Embodiment, enaction, and ecological realism: clarifying theoretical commitments

The embodied framework developed here is situated within a broader landscape of philosophical and theoretical approaches that include enactivism and ecological psychology. While these traditions share a rejection of classical representationalism, important differences remain regarding the status of internal modeling, realism, and the explanatory role of prediction.

Enactivist accounts emphasize sense-making as emerging through autonomous, self-organizing interaction between organism and environment, often resisting explanatory reliance on internal representational content (Varela et al., 1991; Noë, 2004). Ecological approaches, in contrast, stress direct perception of affordances within structured environments and maintain a strong commitment to ecological realism (Gibson, 1979). Both traditions converge in treating perception as constituted through lawful organism–environment relations rather than internal reconstruction.

Recent debates have highlighted tensions between enactivist and predictive processing frameworks, particularly concerning the role of internal generative models and the free energy principle (Di Paolo et al., 2022; Raja et al., 2021; Parr et al., 2024; Sun et al., 2024). Some interpretations of predictive processing remain reformist, retaining representational constructs within hierarchical generative modeling, whereas more radical or eliminativist interpretations emphasize action-oriented regulation and organism–environment coupling.

The position advanced in this paper aligns most closely with an action-oriented, regulation-based interpretation of prediction. Prediction is not treated as the construction of detached internal representations of hidden causes, but as a mechanism for coordinating embodied engagement over time. In this respect, the present account is compatible with ecological realism insofar as environmental structure constrains perceptual organization, while rejecting the claim that perception requires content-rich internal reconstruction.

By explicitly situating the framework within these ongoing debates, the present synthesis aims not to replace enactivist or ecological approaches but to integrate their core insights with contemporary developments in embodied artificial intelligence and predictive learning systems.

## 3 Limitations of disembodied models in cognitive science and artificial intelligence

The embodied account of perception developed in Section 2 provides a principled lens through which to reassess the limitations of disembodied models in both cognitive science and artificial intelligence. If perception emerges through action-dependent regulation, sensorimotor contingencies, and temporally extended interaction, then models that treat perception as an isolated stage of internal signal processing are necessarily incomplete. The shortcomings of disembodied approaches are not merely technical or implementation-related but reflect deeper theoretical assumptions about the nature of perceptual organization.

### 3.1 Classical cognitive models

Classical cognitive models of perception were built on the assumption that perceptual processing could be cleanly separated from cognition and action. Within this framework, perception was treated as an input module responsible for constructing internal representations of the external world, which were then passed to higher cognitive systems for interpretation and decision-making. Although this modular architecture provided analytic clarity and inspired decades of productive research, it struggled to account for the context sensitivity, flexibility, and developmental plasticity of real-world perception.

From the perspective of embodied cognition, the core limitation of classical models lies in their neglect of the constitutive role of action in shaping perceptual experience. By treating action as an output of perception rather than as a co-determining factor, these models fail to explain how perceptual stability is maintained in the face of noisy, ambiguous, and underdetermined sensory input. Empirical research in perceptual psychology demonstrates that perception varies systematically with bodily state, effort, and action capability, effects that cannot be easily accommodated within stimulus-driven or representation-centric accounts (Proffitt, 2006; Witt, 2011). Such findings suggest that perception is scaled to an organism’s possibilities for action, a feature that classical models do not readily capture.

Neuroscientific evidence further challenges the assumption of strict modularity (Fodor, 1983). Contemporary studies reveal extensive bidirectional coupling between sensory and motor systems, with motor planning, prediction, and intention influencing early sensory processing (Gallese et al., 1996). These findings undermine the notion of a unidirectional perceptual pipeline and instead support a view of perception as dynamically intertwined with action and prediction (Rizzolatti and Sinigaglia, 2016; Summerfield and de Lange, 2014). From an embodied standpoint, the failure of classical models is thus not simply that they omit action, but that they misconstrue the basic architecture of perceptual systems by isolating perception from the very processes that give it structure and meaning.

Moreover, classical approaches encounter difficulty in explaining how perceptual organization develops over time. Developmental evidence indicates that perceptual abilities emerge through active exploration and sensorimotor calibration, rather than through the maturation of pre-specified representational mechanisms. Models that emphasize internal reconstruction without accounting for embodied learning struggle to explain why disruptions to early motor experience can have lasting effects on perceptual development. This limitation becomes especially salient when considering neurodevelopmental diversity, where differences in action and embodiment can give rise to divergent perceptual trajectories.

### 3.2 Disembodied artificial intelligence and brittleness

The limitations of disembodied perception models are particularly evident in artificial intelligence, where the consequences of theoretical assumptions can be directly observed in system behavior. Traditional AI perception systems, including many contemporary deep learning architectures, treat perception as a problem of mapping sensory input to internal representations or classifications using large datasets and static input–output relationships. While these approaches have achieved impressive performance in controlled settings, they often exhibit brittleness when confronted with novel contexts, dynamic environments, or tasks requiring active exploration.

From an embodied perspective, the brittleness of disembodied AI systems reflects the absence of sensorimotor grounding. Without the ability to act in the world and observe the sensory consequences of those actions, AI systems lack access to the structured feedback that supports perceptual calibration and self-organization. As a result, perceptual representations learned from passive data remain fragile, overly sensitive to superficial statistical regularities, and poorly suited for generalization beyond the training distribution (LeCun et al., 2015; Lake et al., 2017, 2023; Marcus, 2018; Marcus and Davis, 2019).

Recent critiques emphasize that perception in biological systems is inherently closed-loop, with action shaping sensory input and prediction guiding exploration. Disembodied AI systems, by contrast, operate largely in open-loop regimes, where perception is divorced from action and learning is driven by externally defined objectives rather than intrinsic sensorimotor regularities. This disconnect helps explain why such systems often fail in situations that humans navigate with ease, such as adapting to novel viewpoints, coping with sensory noise, or learning new tasks from minimal experience.

Importantly, the embodied framework developed in Section 2 clarifies that these failures are not simply due to insufficient data or model complexity. Increasing representational capacity alone does not resolve the fundamental problem that perception requires action-dependent structure. Without embodiment, AI systems cannot exploit the regularities that arise from lawful sensorimotor contingencies, nor can they stabilize perception through active sampling and temporal coordination. As a result, perceptual organization remains brittle and context-bound, rather than robust and adaptive.

These limitations have motivated a growing shift within AI toward embodied, interactive, and predictive approaches to perception. However, the persistence of disembodied assumptions in many AI systems underscores the importance of theoretical clarity regarding the nature of perception itself. By situating perception within embodied interaction, the present framework provides a coherent explanation for why disembodied models struggle in both cognitive science and AI, and why embodiment should be treated as a foundational requirement rather than an optional enhancement.

In sum, the critique of disembodied models articulated in this section directly inherits the commitments of embodied cognition outlined in Section 2. If perception is fundamentally enacted, action-dependent, and temporally structured, then models that abstract perception away from embodiment are necessarily limited in their explanatory and practical power. This conclusion sets the stage for the next section, which examines how embodied and self-organizing AI models attempt to overcome these limitations by treating perception as an emergent property of interaction rather than as a static representational problem.

## 4 Emergence of embodied and self-organizing artificial intelligence models

The limitations of disembodied models in cognitive science and artificial intelligence have motivated a growing shift toward approaches that treat perception as an emergent property of embodied interaction rather than as a static representational problem. In contrast to classical architectures that separate perception, cognition, and action into sequential stages, embodied and self-organizing AI models are designed around closed-loop sensorimotor coupling, temporal continuity, and adaptive regulation. These models align closely with the embodied account of perception articulated in Section 2, treating action not as an output of perception but as a constitutive factor in perceptual organization.

This shift reflects a broader recognition that perceptual systems must actively structure their sensory input in order to learn and generalize. Rather than attempting to infer the structure of the world from passive data, embodied AI systems exploit the regularities that arise from lawful interaction between an agent’s body, its actions, and the environment. Perception, within these systems, emerges as a process of ongoing calibration and self-organization driven by feedback, prediction, and exploration. This approach reframes intelligence not as the manipulation of internal symbols or representations, but as the capacity to regulate interaction with the world in adaptive and temporally coherent ways.

### 4.1 Embodiment as a computational resource

One of the central insights of embodied AI is that physical embodiment can function as a computational resource rather than a constraint. Research in robotics and embodied intelligence demonstrates that bodily morphology, material properties, and sensorimotor layout can simplify perceptual and control problems by embedding structure directly into the dynamics of interaction. This principle, often referred to as morphological computation, highlights how aspects of perception and coordination can be offloaded from central processing to the physical coupling between body and environment (Pfeifer and Bongard, 2006; Hauser et al., 2011; Liu et al., 2025).

Embodied agents exploit their physical form to generate structured sensory input, reducing the need for exhaustive internal modeling. For example, compliant bodies and sensorimotor synergies can stabilize interaction patterns, allowing perceptual regularities to emerge naturally through movement. Empirical work in robotics shows that such systems often display greater robustness, adaptability, and energy efficiency than disembodied counterparts that rely on centralized control and abstract representations (Pfeifer et al., 2014; Levine et al., 2023). These findings parallel observations in biological systems, where perception is shaped by bodily constraints and action capabilities rather than by stimulus encoding alone.

From the perspective developed in this paper, embodiment contributes directly to perceptual organization by constraining the space of possible interactions. By limiting and structuring how an agent can act, embodiment reduces perceptual ambiguity and supports the emergence of stable sensorimotor contingencies. In this sense, embodiment is not merely a means of acting on perceptual representations but a foundational component of perception itself. Artificial systems that lack such constraints must compensate through increased representational complexity, often with limited success, reinforcing the importance of embodiment for perceptual learning and generalization.

It is important to distinguish between different forms of embodiment in artificial systems. In physical robotics, embodiment refers to agents whose morphology, materials, and sensorimotor dynamics are realized in real-world physical interaction. In such systems, mechanical compliance, friction, inertia, and environmental coupling directly shape perceptual input and learning dynamics (Pfeifer and Bongard, 2006; Hauser et al., 2011). By contrast, many contemporary “embodied” AI models operate in simulated environments, where embodiment is instantiated through virtual physics engines and parameterized sensorimotor interfaces. Although simulation-based agents lack material constraints in the strict physical sense, they nevertheless engage in closed-loop action–perception cycles that generate structured sensory trajectories over time (Hafner et al., 2023; Levine et al., 2023).

Both forms of embodiment provide advantages over disembodied feedforward architectures, but they differ in the degree to which morphology and environmental resistance function as intrinsic computational constraints. Physical embodiment embeds structure directly in material interaction, whereas simulation embodiment embeds structure in modeled dynamics. Recognizing this distinction clarifies that embodiment is not binary but exists along a continuum of sensorimotor coupling, with implications for how perceptual self-organization emerges in artificial systems.

### 4.2 Self-organization and learning through interaction

A second defining feature of contemporary embodied AI models is their reliance on self-organization rather than explicit programming or centralized control. Self-organizing systems develop perceptual and behavioral structure through local interactions, feedback loops, and adaptive processes that unfold over time. Rather than encoding perceptual categories or action policies in advance, these systems allow structure to emerge through repeated engagement with the environment (Kelso, 1995; Haken, 1983).

In developmental robotics and embodied reinforcement learning, agents are often initialized with minimal prior knowledge and allowed to explore their environment through intrinsic motivation or predictive objectives. Through sensorimotor exploration, perceptual regularities and coordinated behaviors gradually emerge without direct supervision. Studies demonstrate that such agents can develop stable perceptual categories, action repertoires, and predictive models as a consequence of interaction, mirroring key aspects of human perceptual development (Smith and Gasser, 2005; Oudeyer et al., 2007; Hafner et al., 2023, 2025; Hao et al., 2023).

**Figure 2:**
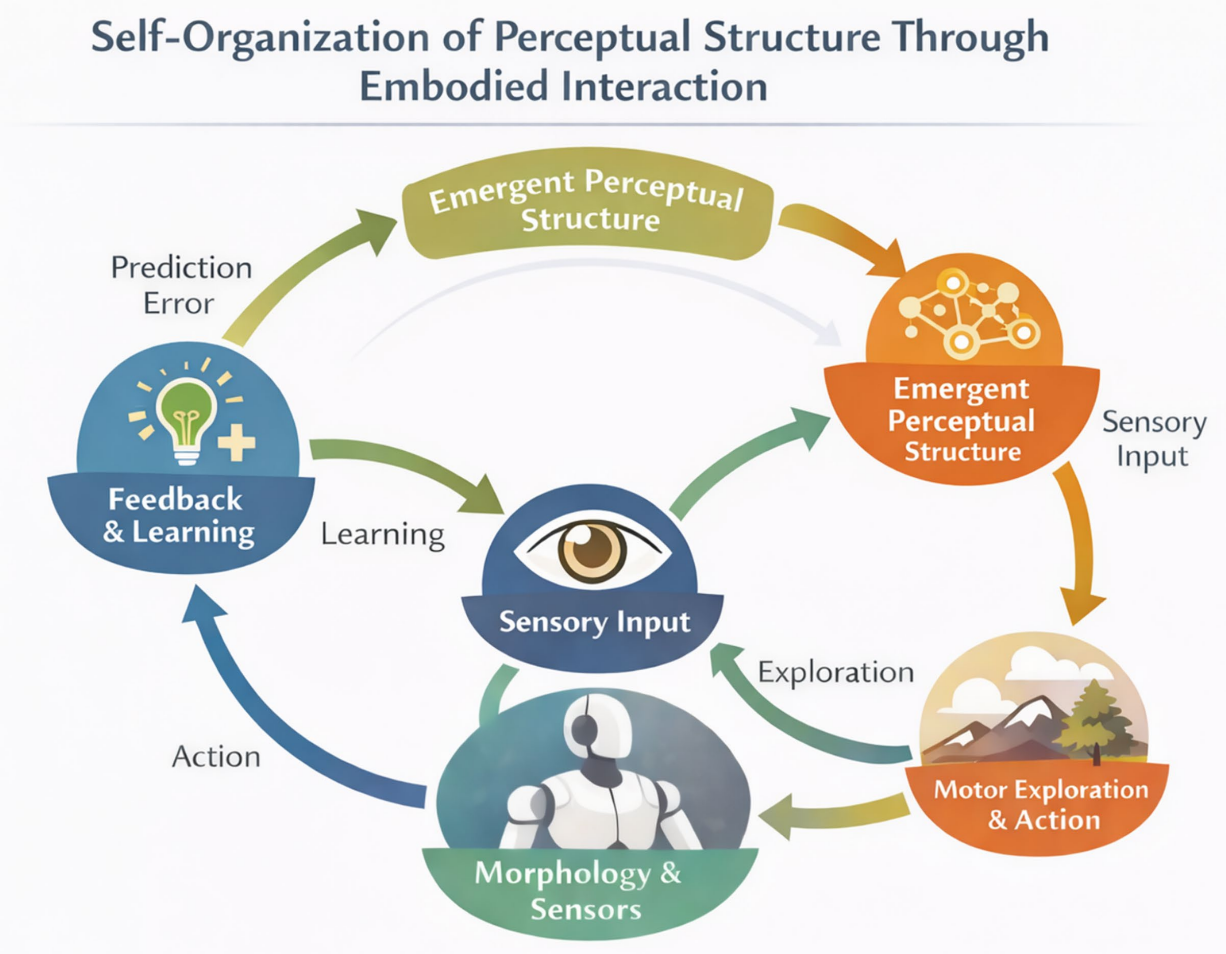
Self-organization of perceptual structure through embodied interaction. This schematic illustrates how perceptual organization emerges through continuous interaction among an agent’s morphology, sensorimotor exploration, and environmental feedback. Bodily constraints and sensor layout shape patterns of action, which in turn generate structured sensory input. Learning is driven by feedback and prediction error, allowing stable perceptual categories and coordinated behaviors to emerge over time. Perception is not pre-specified or centrally controlled but self-organizes through repeated embodied engagement with the environment.

Crucially, learning in these systems is driven by the agent’s own actions and their sensory consequences. Perceptual organization arises not from exposure to large datasets, but from the structured feedback generated by closed-loop interaction. This learning process aligns closely with the embodied account of perception outlined in Section 2, in which perceptual meaning emerges through mastery of sensorimotor contingencies and temporal coordination. Self-organization thus provides a mechanistic bridge between embodied interaction and perceptual stability, explaining how coherent perception can arise without pre-specified representations (Figure 2).

Importantly, self-organizing embodied AI models emphasize the temporal dimension of perception. Perceptual structure emerges gradually through interaction across multiple timescales, from moment-to-moment sensorimotor loops to longer-term learning dynamics. This temporal extension allows agents to integrate past actions with present sensory input and future expectations, supporting perceptual continuity in dynamic environments. Without such temporal integration, perceptual systems remain reactive and fragile, a limitation characteristic of many disembodied AI models.

Taken together, embodiment and self-organization provide a coherent alternative to representational approaches to artificial perception. By grounding perception in action, bodily constraints, and temporally extended interaction, embodied AI models demonstrate how perceptual organization can emerge naturally through learning rather than being imposed through design. This approach not only addresses the brittleness of disembodied systems but also offers a formal framework for exploring perception as a process of interaction, setting the stage for the integration of predictive processing and temporal coordination examined in the next section.

Despite their promise, world-model-based and self-organizing AI architectures face important limitations. Current world models often rely on learned latent representations that approximate environmental dynamics within constrained domains. While these systems demonstrate impressive performance in simulated or semi-structured environments (Hafner et al., 2023, 2025; Silver et al., 2024; Hao et al., 2023), their generalization remains bounded by training distributions, architectural priors, and computational scalability. Learned latent spaces may capture regularities efficiently but can also entrench inductive biases that limit transfer across radically different contexts.

Moreover, simulation-based embodiment lacks the full richness of biological interaction, including metabolic constraints, developmental plasticity, and sociocultural embedding. Even physically embodied robots typically operate in simplified environments compared to biological organisms. As such, contemporary world-model approaches should be understood as approximations of embodied perceptual learning rather than full instantiations of organism–environment self-organization.

Recognizing these limitations reinforces the theoretical claim advanced in this paper: embodiment functions as a generative constraint on perception, but its computational realization remains an open research frontier. Continued integration of robotics, developmental learning paradigms, and temporally structured predictive architectures will be necessary to approach the robustness observed in biological systems.

## 5 Perception as predictive, action-dependent inference

The embodied and self-organizing account of perception developed in the preceding sections provides a natural foundation for predictive processing models, while also placing important constraints on how prediction should be understood in perceptual systems. Predictive approaches propose that perception involves the continuous generation of expectations about sensory input, with discrepancies between predicted and actual input driving learning and adaptation. When integrated with embodiment, however, prediction is not a purely inferential operation performed on sensory data, but an action-dependent process through which agents regulate their engagement with the environment over time.

### 5.1 Predictive processing in biological systems

Predictive processing models in neuroscience emphasize that the brain actively anticipates sensory input and updates its expectations based on prediction error. Rather than passively registering stimuli, perceptual systems are understood to operate prospectively, using prior experience to guide sampling of the environment and to stabilize perception in the face of noise and ambiguity (Friston, 2010; Clark, 2013). Importantly, contemporary formulations increasingly recognize that prediction is inseparable from action. Organisms do not merely update internal models in response to error; they act in ways that structure sensory input to confirm, refine, or disambiguate their expectations.

Empirical evidence supports this action-oriented view of prediction. Motor planning and efference signals influence early sensory processing, indicating that perception is shaped by anticipated action outcomes rather than by stimulus input alone (Wolpert et al., 1995; Summerfield and de Lange, 2014). From an embodied perspective, prediction functions as a mechanism for coordinating sensorimotor engagement, allowing agents to select actions that reduce uncertainty by generating informative sensory consequences. Perceptual experience thus reflects not only what is sensed, but how the organism is prepared to act.

This interpretation resolves a potential tension between predictive processing and embodied cognition. If prediction is treated as a disembodied inferential mechanism operating on internal representations, it risks reproducing the limitations of classical perceptual models. When prediction is instead understood as action-dependent regulation, it becomes compatible with the view that perception is enacted through interaction. Predictive signals guide exploration, while action shapes the sensory evidence available for updating expectations. Perception, in this sense, is neither purely bottom-up nor top-down, but emerges through the continuous coupling of prediction and action.

### 5.2 Predictive and active inference in artificial intelligence

Parallel developments in artificial intelligence reflect a similar shift toward action-centered predictive models. In active inference and world-model-based learning, artificial agents are designed to minimize prediction error not only by updating internal models, but by selecting actions that structure sensory input in advantageous ways (Friston et al., 2017; Pezzulo et al., 2024; Hafner et al., 2023, 2025; Hao et al., 2023; Qiao et al., 2024; Schiewer et al., 2024). These systems treat perception and action as components of a single adaptive loop, rather than as sequential stages in a processing pipeline.

Embodied AI models implementing predictive or active inference frameworks demonstrate that perception becomes more robust when agents can explore, test, and refine predictions through interaction. By acting in the environment, agents generate structured sensory input that constrains learning and supports the emergence of coherent perceptual organization. In contrast, predictive models that lack embodiment remain underconstrained, relying heavily on priors learned from static data and exhibiting limited generalization. This distinction underscores the importance of embodiment for grounding prediction in lawful sensorimotor contingencies.

**Figure 3:**
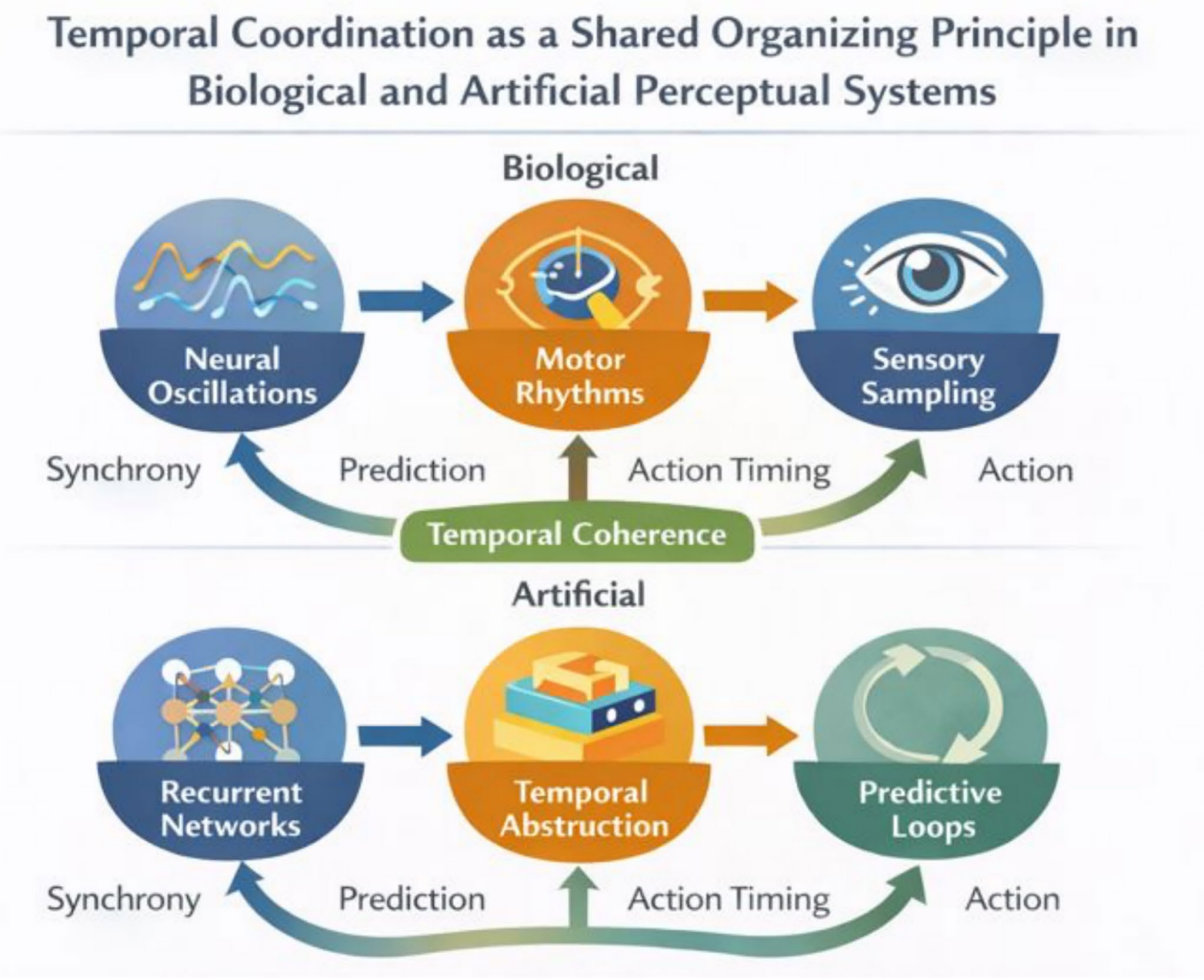
Predictive, action-dependent perception in embodied biological and artificial systems. This figure illustrates perception as a closed-loop process in which predictive signals and action jointly regulate sensory input over time. In both biological and artificial agents, predictions guide action, action structures sensory input, and prediction error drives learning and adaptation. Perceptual organization emerges from this continuous interaction rather than from passive inference or static representation, highlighting the inseparability of perception, action, and prediction in embodied systems.

From the perspective advanced in this paper, predictive processing should be understood as one mechanism through which embodied systems achieve perceptual stability. Prediction does not replace sensorimotor interaction but depends on it. Action provides the means by which predictions are evaluated, refined, and stabilized across time. Artificial systems that integrate prediction with embodied interaction thus inherit the advantages of biological perceptual systems, including adaptability, resilience to noise, and sensitivity to context. Figure 3 schematically depicts this predictive, action-dependent loop in both biological and artificial systems, illustrating how predictions guide action, action structures sensory input, and prediction error drives learning through continuous closed-loop interaction.

Critically, predictive processing frameworks also emphasize the temporal dimension of perception. Predictions unfold across multiple timescales, from moment-to-moment sensorimotor anticipation to longer-term learning and adaptation. Embodied agents must integrate information over time to maintain perceptual continuity in dynamic environments. Without such temporal integration, prediction remains reactive and fragile, a limitation that parallels the brittleness observed in disembodied AI systems.

By situating predictive processing within an embodied and self-organizing framework, this section clarifies how prediction contributes to perceptual organization without reverting to representationalist assumptions. Perception emerges as an active, temporally extended process through which agents regulate their interaction with the world. This interpretation sets the stage for the next section, which examines temporal structure and rhythmic coordination as fundamental organizing principles that support prediction, action, and perceptual coherence across biological and artificial systems.

### 5.3 Clarifying the status of representation in predictive processing

The integration of predictive processing within an embodied framework requires conceptual clarification regarding the status of representation. Predictive models are often interpreted as positing hierarchical internal generative models that encode probabilistic representations of hidden environmental causes (Friston, 2010; Clark, 2013). Such reformist interpretations preserve representational language while updating its computational implementation.

However, alternative interpretations emphasize that prediction need not entail content-rich internal representations in the classical sense. Instead, predictive dynamics can be understood as regulation of organism–environment coupling through action-dependent error minimization, without committing to representational reconstruction as an explanatory primitive (Di Paolo et al., 2022; Raja et al., 2021). On this view, generative structure reflects constraints on interaction rather than internally stored descriptions of the world.

The position adopted in this paper aligns with this regulation-centered interpretation. Prediction is treated as enacted coordination across sensorimotor loops rather than as detached modeling of hidden states. Neural or computational generative architectures may be formally described as models, but their functional role is to guide embodied engagement and maintain adaptive coupling over time. Perception thus remains grounded in interaction, even when described using predictive or generative terminology.

This clarification avoids a false dichotomy between embodiment and predictive processing. Representational vocabulary may serve heuristic or mathematical purposes, but explanatory priority is assigned to dynamic interaction, temporal regulation, and lawful organism–environment structure. In this sense, predictive processing is incorporated into the present framework without reintroducing the representationalism that embodied cognition originally sought to transcend.

## 6 Temporal structure, rhythm, and coordination

The embodied and predictive accounts of perception developed in the preceding sections converge on a critical organizing dimension that has often been treated as secondary in perceptual theory: time. Perception does not occur instantaneously or in isolation but unfolds over multiple temporal scales through the coordination of sensation, action, and prediction. From an embodied perspective, temporal structure is not merely a property of neural processing but emerges from rhythmic interaction between an agent and its environment. Perceptual coherence depends on the alignment of sensory sampling, motor activity, and predictive expectations across time, making temporal coordination a foundational component of perceptual organization.

### 6.1 Temporal dynamics in biological perception

Biological perceptual systems are intrinsically rhythmic. Neural activity is organized by oscillatory processes that structure the timing of sensory sampling, information integration, and action preparation. Research in systems neuroscience demonstrates that low- and high-frequency neural rhythms coordinate activity across distributed networks, shaping when sensory information is amplified, suppressed, or integrated (Buzsáki, 2006; Schroeder and Lakatos, 2009). These temporal dynamics are not epiphenomenal but play a functional role in perception by aligning internal processing with external events.

Crucially, perceptual timing is tightly coupled to action. Movements such as eye saccades, locomotion, and exploratory gestures introduce rhythmic structure into sensory input, creating predictable patterns that perceptual systems exploit. Empirical studies show that motor rhythms influence perceptual segmentation and multisensory integration, indicating that perception is organized around action-generated temporal regularities rather than continuous stimulus streams (Large and Snyder, 2009; Ivry and Schlerf, 2007; Leisman et al., 2026). From an embodied standpoint, temporal coordination between movement and sensation provides a scaffold for perceptual stability, allowing organisms to anticipate and integrate sensory events over time.

Developmental evidence further underscores the importance of temporal structure. The maturation of perceptual abilities is closely linked to the development of rhythmic motor activity and sensorimotor timing. Disruptions to temporal coordination, whether due to neurological injury or atypical development, are associated with impairments in perceptual integration, learning, and action planning. These findings suggest that timing is not an auxiliary feature of perception but a core organizing principle that shapes how perceptual meaning emerges through interaction.

### 6.2 Temporal coordination in artificial intelligence systems

Recent advances in artificial intelligence increasingly reflect the recognition that temporal structure is essential for robust perception. Disembodied models that rely on static input–output mappings often struggle to maintain perceptual coherence in dynamic environments, in part because they lack mechanisms for integrating information across time. In contrast, embodied and recurrent architectures explicitly model temporal dependencies, allowing perceptual representations to evolve in response to ongoing interaction (Jaeger and Haas, 2004; Kietzmann et al., 2019).

World-model-based and predictive AI systems incorporate temporal abstraction as a central design principle. By learning latent dynamics that span multiple timescales, these systems can anticipate future sensory states and coordinate action accordingly. Importantly, temporal coherence in such models is not imposed externally but emerges through interaction, as agents learn how their actions influence sensory trajectories over time (Hafner et al., 2023, 2025; Wang et al., 2024; Hao et al., 2023). This mirrors biological perception, where timing is shaped by the regularities of embodied engagement rather than by isolated sensory events.

**Figure 4:**
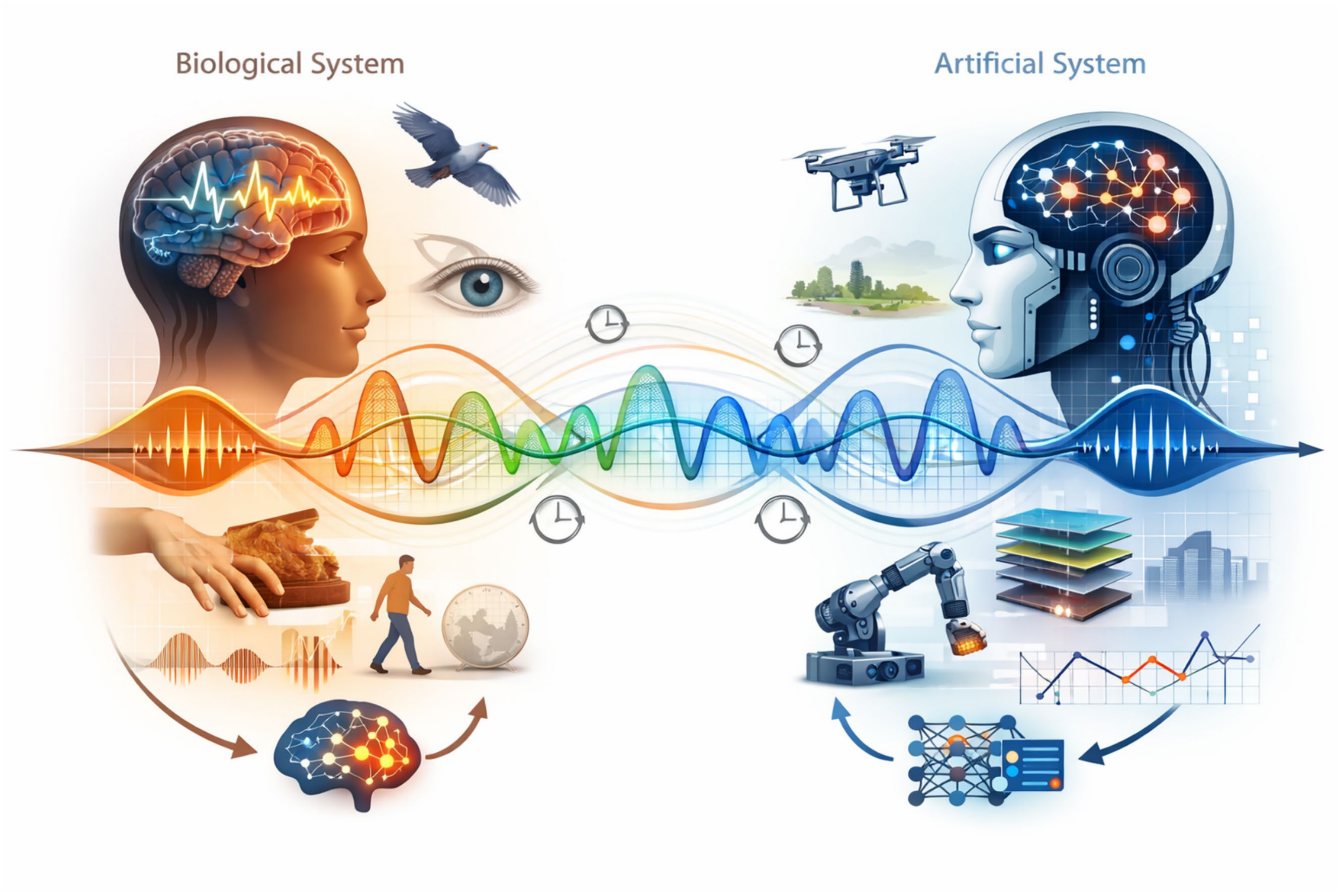
Temporal coordination as a unifying organizing principle in biological and artificial perceptual systems. This schematic illustrates how temporal structure links sensation, action, and prediction across multiple timescales. In biological systems, rhythmic neural activity and movement patterns coordinate sensory sampling and perceptual integration. In artificial systems, recurrent and world-model-based architectures enable temporal abstraction and predictive regulation. In both cases, perceptual coherence emerges from the alignment of sensory input, action, and prediction over time, highlighting temporal coordination as a foundational constraint on perception.

From the perspective developed in this paper, temporal coordination serves as the glue that binds embodiment and prediction into a coherent perceptual process. Prediction requires temporal structure to generate expectations, while action provides the rhythmic sampling necessary to test and refine those expectations. Artificial systems that lack this temporal grounding remain reactive and brittle, whereas systems that integrate temporal dynamics with embodiment can achieve perceptual continuity and adaptability in complex environments. Figure 4 schematically illustrates how temporal coordination functions as a unifying organizing principle across biological and artificial perceptual systems, showing how rhythmic sensory sampling, action, and predictive regulation align across multiple timescales to support perceptual coherence.

Taken together, the evidence reviewed in this section supports the view that perception is fundamentally a temporally extended process. Embodiment provides the means by which agents generate structured sensory input, prediction supplies anticipatory regulation, and temporal coordination integrates these processes into stable perceptual experience. Recognizing time as a central organizing dimension allows perceptual science to bridge biological and artificial systems within a common explanatory framework, setting the stage for the implications of this view for perceptual theory, AI design, and autism spectrum disorder examined in the subsequent sections.

## 7 Implications for perceptual science and AI design

Reconceptualizing perception as an embodied, predictive, and temporally structured process has substantive implications for both perceptual science and the design of artificial intelligence systems. Across the preceding sections, perception has been framed not as the passive encoding of sensory input or the inference of hidden causes, but as an emergent process of regulation that unfolds through action-dependent interaction over time. This shift calls for a reassessment of how perceptual phenomena are theorized, studied, and modeled across disciplines.

For perceptual science, the embodied framework challenges the long-standing tendency to privilege stimulus-based explanations and laboratory paradigms that minimize movement, interaction, and temporal structure. While such approaches have yielded valuable insights into sensory processing, they risk obscuring the constitutive role of action in perceptual organization. If perception depends on sensorimotor contingencies and temporal coordination, then experimental designs that constrain or eliminate action may capture only a partial and potentially misleading picture of perceptual function. An embodied perspective thus motivates a greater emphasis on paradigms that incorporate active exploration, dynamic environments, and real-time interaction, allowing perceptual processes to be studied in conditions that more closely approximate their natural operating context.

This reframing also has implications for how perceptual stability, ambiguity, and variability are understood. Within an embodied account, perceptual constancies are not achieved through increasingly accurate internal representations but through reliable patterns of engagement that stabilize sensory input over time. Variability in perception, whether across individuals or contexts, is therefore not necessarily noise or error but may reflect differences in embodiment, action strategies, and interaction histories. This view aligns with growing interest in perceptual diversity and context dependence, encouraging theories that account for how perception adapts to bodily constraints and environmental demands rather than abstracting away from them.

The integration of predictive processing within an embodied framework further reshapes perceptual theory by emphasizing regulation over inference. Prediction, when grounded in action, functions to coordinate engagement with the environment rather than to reconstruct its latent structure. This perspective helps reconcile predictive accounts with empirical findings showing that perception is prospective, task-dependent, and shaped by movement and timing. For perceptual science, this suggests that prediction should be studied not only as a computational mechanism but as a process embedded in ongoing interaction, sensitive to temporal structure and bodily dynamics.

For artificial intelligence, the implications are equally consequential. The persistent brittleness of disembodied AI systems underscores the limitations of treating perception as a static mapping from input to representation. The embodied framework developed here suggests that robust artificial perception requires systems capable of acting in the world, generating structured sensory input, and learning through temporally extended interaction. Embodiment, in this context, should be understood not merely as the addition of sensors and actuators, but as the imposition of constraints that shape learning, prediction, and perceptual organization.

Designing AI systems around embodied, self-organizing principles shifts the focus from representational complexity to interactional competence. Rather than attempting to encode exhaustive models of the environment, embodied AI systems can exploit the regularities that arise from lawful sensorimotor coupling, allowing perceptual structure to emerge through use. This approach supports adaptability and generalization by grounding perception in action-dependent feedback rather than in static datasets. It also highlights the importance of temporal abstraction and rhythm, as artificial agents must integrate information across time to maintain perceptual coherence in dynamic settings.

Importantly, the convergence between perceptual science and embodied AI creates opportunities for mutual constraint and cross-fertilization. AI systems can serve as formal testbeds for embodied perceptual theories, allowing researchers to manipulate embodiment, prediction, and temporal structure in ways that are difficult or impossible in biological systems. Conversely, empirical findings from perceptual science can inform the design of artificial agents that learn and perceive in more human-like and context-sensitive ways. This reciprocal relationship supports a more unified science of perception that spans biological and artificial domains without reducing one to the other.

Taken together, the implications outlined in this section point toward a shift in both theory and practice. Understanding perception as embodied, predictive, and temporally coordinated encourages perceptual science to move beyond static, representation-centric models and motivates AI design approaches that prioritize interaction, learning, and regulation over inference alone. This shift provides the conceptual groundwork for extending the framework to neurodevelopmental diversity, where differences in embodiment, timing, and prediction offer a powerful lens for understanding perceptual variation, as examined in the following section on autism spectrum disorder.

## 8 Implications for autism Spectrum disorder and AI-informed perceptual frameworks

The embodied, predictive, and temporally structured account of perception developed in this paper has particular relevance for understanding autism spectrum disorder (ASD), where differences in sensory experience, motor coordination, and perceptual integration are widely reported across development. Traditionally, many theoretical accounts of autism have focused on higher-order cognitive or social deficits, often treating sensory and motor features as secondary or peripheral. However, a growing body of empirical work indicates that atypical sensory processing and action patterns are core, early-emerging features of autism that shape perceptual experience and developmental trajectories (Tavassoli et al., 2020; Thye et al., 2022; Green et al., 2023; Botha et al., 2024). From an embodied perceptual perspective, these findings suggest that autism may be more productively understood as a difference in how perceptual systems self-organize through interaction rather than as a failure of abstract cognition.

### 8.1 Autism as a difference in embodied perceptual organization

Large-scale behavioral, developmental, and neurophysiological studies increasingly converge on the view that autistic perception is characterized by differences in sensory responsivity, sensorimotor coordination, and perceptual integration across modalities. These differences include heightened sensitivity to sensory input, reduced contextual modulation, and variability in multisensory binding, all of which are evident early in development and persist across the lifespan (Tavassoli et al., 2020; Meilleur et al., 2020). Importantly, such features are not uniformly impairing but reflect distinct patterns of perceptual organization that interact with environmental demands and individual action strategies.

From an embodied cognition standpoint, these perceptual differences can be interpreted as arising from variations in sensorimotor contingencies and action-dependent calibration. If perceptual meaning emerges through mastery of lawful relationships between action and sensation, then differences in movement patterns, postural control, or exploratory behavior will necessarily shape perceptual organization. Longitudinal studies show that motor differences and altered movement variability are detectable in infancy among children later diagnosed with autism, often preceding the emergence of social-communicative differences (Iverson et al., 2021; West et al., 2022). These findings support a developmental cascade model in which early differences in embodiment influence how perception self-organizes over time.

Reframing autism in terms of embodied perceptual organization shifts emphasis away from deficit-based explanations toward a process-oriented account of perceptual diversity. Rather than asking why autistic individuals fail to perceive the world in typical ways, the embodied framework asks how different patterns of interaction give rise to distinct but internally coherent perceptual experiences. This perspective aligns with contemporary neurodiversity-informed approaches that emphasize variability, adaptation, and lived experience rather than normative benchmarks of perceptual function (Happé and Frith, 2020; Lombardo et al., 2022).

Recent enactive and ecological accounts of autism further support this interaction-centered interpretation. De Jaegher (2013) argues that autistic experience should be understood in terms of differences in participatory sense-making, emphasizing how meaning emerges through embodied social interaction rather than through internal cognitive deficit. Expanding this perspective, De Jaegher (2023) highlights the importance of inviting and sustaining interactional engagement, suggesting that differences in autism may reflect distinct dynamics of coordination rather than impaired mental representation.

Similarly, ecological-enactive analyses propose that autism involves alterations in how affordative space is structured and navigated through action (Cadena-Alvear and Gastelum-Vargas, 2024), as well as differences in organism–environment coupling that influence perceptual salience and behavioral regulation (Nešić, 2023). These approaches converge with the framework advanced here by treating perception not as a detached internal process but as a form of embodied participation in structured environments.

Integrating these perspectives strengthens the claim that autistic perception reflects differences in self-organizing interaction rather than representational deficit. The present account extends this enactive and ecological literature by linking interactional sense-making with predictive regulation and temporally structured embodiment, thereby situating autism within a broader cross-disciplinary synthesis that includes artificial intelligence and formal models of perceptual self-organization.

### 8.2 Predictive regulation, sensory precision, and action in autism

Predictive processing accounts of autism provide an additional layer of insight into perceptual differences by focusing on how sensory evidence and prior expectations are weighted during perception. Recent empirical work suggests that autistic individuals may assign greater precision to sensory input relative to prior expectations, leading to heightened perceptual detail, reduced contextual influence, and increased sensitivity to variability in the environment (Lawson et al., 2021; Palmer et al., 2023; Karvelis et al., 2022). Importantly, these findings do not imply a deficit in prediction per se, but rather a difference in how prediction error is regulated.

When integrated with an embodied framework, altered predictive regulation in autism can be understood as inseparable from differences in action-dependent sensory sampling. Prediction is not an abstract inferential process operating independently of behavior; it is calibrated through movement and exploration. If sensorimotor engagement is constrained, repetitive, or temporally dysregulated, predictive models may stabilize locally while remaining less flexible globally. This interpretation helps explain why repetitive behaviors and restricted action patterns in autism may function to reduce perceptual uncertainty by stabilizing sensorimotor contingencies, rather than reflecting simple behavioral rigidity (Kapp et al., 2020; Lawson et al., 2022; Kapp, 2020).

From this perspective, perception, action, and prediction form a tightly coupled system that adapts differently in autism. Differences in predictive regulation are thus not isolated cognitive features but emerge from the interaction between sensory precision, movement patterns, and environmental structure. This embodied-predictive synthesis provides a coherent account of why autistic perception often emphasizes detail, immediacy, and local regularities, while de-emphasizing global contextual integration.

Importantly, the predictive processing literature in autism is heterogeneous, and empirical findings do not uniformly support a single alteration in sensory precision or prior weighting. While several studies suggest increased precision of sensory evidence or altered volatility estimation (Lawson et al., 2021; Palmer et al., 2023), other work reports context-dependent effects, developmental variability, and task-specific modulation. Differences in predictive regulation appear to vary across individuals, sensory modalities, and developmental stages. Accordingly, the present framework does not posit a uniform predictive profile in autism but instead interprets altered precision weighting as one dimension along which embodied perceptual organization may diverge. Future empirical work integrating movement measures, longitudinal design, and multimodal prediction paradigms will be necessary to clarify how predictive regulation interacts with embodiment across the autism spectrum.

### 8.3 Temporal coordination and perceptual integration in autism

Temporal structure plays a central role in perceptual organization, particularly in multisensory and social contexts. A growing literature indicates that autistic individuals often show differences in temporal processing, including broader multisensory temporal binding windows, altered rhythmic entrainment, and variability in perceptual timing across modalities (Foss-Feig et al., 2020; Noel et al., 2021; Meilleur et al., 2020). These differences are evident not only in social perception but also in basic sensory and motor timing, suggesting a domain-general alteration in temporal coordination.

Within the embodied framework developed in this paper, such findings point to time as a critical axis along which perceptual self-organization diverges in autism. If perceptual coherence depends on the alignment of action, sensation, and prediction over time, then differences in temporal coordination will necessarily affect how perceptual meaning emerges. Longitudinal evidence indicates that early differences in sensorimotor timing are predictive of later social-communication outcomes, supporting the view that temporal coordination plays a foundational role in shaping perceptual and developmental trajectories (Woynaroski et al., 2021).

This interpretation challenges accounts that locate social perceptual differences in autism primarily in social motivation or conceptual understanding. Instead, it suggests that social perception depends on the ability to synchronize perceptual predictions with the actions of others, a process that is fundamentally embodied and temporally structured. Differences in timing and rhythm thus have cascading effects on perception across domains, reinforcing the need for process-oriented explanations.

As with predictive precision, evidence concerning temporal processing differences in autism reflects considerable heterogeneity. Broader temporal binding windows and altered rhythmic entrainment have been observed in multiple studies (Foss-Feig et al., 2020; Noel et al., 2021), yet effect sizes vary and are influenced by task demands, age, and sensory modality. Some individuals show intact or even enhanced performance under specific temporal constraints. The embodied account advanced here, therefore, treats temporal coordination differences not as universal impairments but as dimensions of variability in how sensorimotor systems align action, sensation, and prediction across development. Recognizing this variability is essential for avoiding overgeneralization and for maintaining conceptual compatibility with dimensional and neurodiversity-informed models of autism (Lombardo et al., 2022).

### 8.4 Embodied AI models as formal testbeds for autism-relevant perceptual processes

Embodied AI systems offer a unique opportunity to formalize and experimentally probe the perceptual processes implicated in autism. Recent advances in world-model-based learning, active inference, and embodied reinforcement learning demonstrate that perceptual organization emerges from the interaction between sensorimotor constraints, predictive regulation, and temporal integration (Hafner et al., 2023; Levine et al., 2023; Silver et al., 2024). By systematically varying parameters such as sensory precision, action variability, and temporal integration windows, AI models can generate a range of perceptual profiles without invoking categorical impairments.

Such models align closely with contemporary autism research emphasizing dimensional variability and heterogeneity rather than unitary deficit accounts (Lombardo et al., 2022). Importantly, embodied AI allows researchers to examine developmental trajectories by simulating how early differences in interaction can give rise to stable but divergent perceptual organizations over time. This capacity mirrors empirical findings in autism showing that early sensorimotor differences can cascade into long-term differences in perception, learning, and engagement.

While artificial systems necessarily abstract away biological and experiential complexity, their value lies in providing formal, manipulable testbeds for theory refinement. Within the framework advanced here, embodied AI does not serve as a model of autism itself but as a tool for exploring how perceptual self-organization depends on interaction, prediction, and timing. This distinction is critical for avoiding reductive interpretations and for maintaining ethical and conceptual clarity.

### 8.5 Implications for neurodiversity-informed perceptual support

Although the present paper does not propose clinical interventions, the embodied perceptual framework outlined here has implications for how supportive technologies and environments might be conceptualized. Rather than targeting discrete behaviors or skills, an interaction-centered approach emphasizes supporting perceptual coherence by aligning sensory input, action possibilities, and temporal structure with individual perceptual styles. Recent work in human-AI interaction suggests that adaptive systems that respond dynamically to user movement, timing, and sensory preferences can support engagement without imposing normative behavioral expectations (Richer et al., 2022; D’Mello et al., 2023).

From a neurodiversity-affirming perspective, such approaches prioritize accommodation and mutual adaptation rather than normalization. Embodied AI systems, when designed responsibly and in collaboration with autistic individuals, may serve as mediating structures that support perceptual regulation through interaction rather than instruction. This orientation is consistent with the broader shift advocated throughout this paper, in which perception is understood as a process of ongoing coordination rather than a fixed capacity to be corrected.

### 8.6 Toward a process-oriented understanding of autism and perception

Integrating autism research with embodied cognition and AI-driven perceptual models supports a shift from static, deficit-oriented explanations toward process-oriented accounts of neurodevelopmental diversity. Within this framework, autism emerges as a difference in how perceptual systems self-organize through embodied interaction, predictive regulation, and temporal coordination across development. This view accommodates both the heterogeneity of autistic experience and the stability of individual perceptual styles over time.

By grounding perception in interaction rather than abstraction, the framework advanced here provides a coherent explanatory space in which biological diversity and artificial intelligence can be jointly examined. Autism, in this context, is not a failure of perception but a distinct mode of perceptual organization shaped by the dynamics of embodiment, prediction, and time. This process-oriented perspective sets the stage for the integrative discussion and future directions that follow.

## 9 Discussion

The present paper advances a unified theoretical framework in which perception is understood as an embodied, predictive, and self-organizing process that unfolds through temporally structured interaction between agents and their environments. Across perceptual science, neuroscience, artificial intelligence, and autism research, converging lines of evidence increasingly challenge representational accounts that treat perception as the passive reconstruction of sensory input or as an inferential stage preceding action. Instead, perception emerges as an active process of regulation, shaped by bodily constraints, sensorimotor contingencies, predictive dynamics, and temporal coordination.

Sections 2 through 6 developed the core theoretical commitments of this framework. Embodied cognition reframes perception as enacted rather than encoded, emphasizing that perceptual meaning arises through skilled engagement with the environment rather than through internal depiction alone. Sensorimotor contingency theory provides a formal account of how perceptual qualities depend on lawful relations between action and sensation, while self-organizing AI models demonstrate how perceptual structure can emerge through interaction without explicit representational specification. Predictive processing, when grounded in embodiment, contributes anticipatory regulation that guides action and stabilizes perception, and temporal coordination integrates these processes across multiple timescales. Taken together, these elements form a coherent account of perception as an emergent, interaction-centered phenomenon.

A central implication of this synthesis is that perceptual stability does not require increasingly detailed internal models of the world. Instead, stability arises through reliable patterns of engagement that constrain sensory variability over time. This perspective resolves long-standing issues in perceptual theory related to underdetermination and ambiguity by shifting explanatory emphasis from inference to interaction. Perception becomes reliable not because the system reconstructs the world accurately, but because it can act in ways that generate predictable and informative sensory consequences. This interactional view aligns closely with empirical findings showing that perception is modulated by action capabilities, bodily state, and timing, and that sensory processing is deeply intertwined with motor planning and prediction.

The framework also clarifies why disembodied models in both cognitive science and artificial intelligence exhibit systematic limitations. Classical modular accounts isolate perception from action and development, while disembodied AI systems rely on passive data and static mappings that lack sensorimotor grounding. From the embodied perspective advanced here, these approaches fail not due to insufficient computational power or data, but because they abstract perception away from the very processes that give it structure. Embodied and self-organizing AI models, by contrast, demonstrate that perception can emerge robustly through closed-loop interaction, offering formal support for embodied theories and providing tools for exploring perceptual organization in controlled yet dynamic settings.

Extending this framework to autism spectrum disorder highlights its explanatory breadth. Rather than conceptualizing autism primarily in terms of deficits in social cognition or abstract representation, the embodied account situates autistic perception within a process-oriented developmental context. Empirical evidence reviewed in Section 8 indicates that sensory differences, motor variability, altered predictive regulation, and differences in temporal coordination are core features of autism that emerge early and shape perceptual experience across the lifespan. From an embodied-predictive standpoint, these features reflect differences in how perceptual systems self-organize through interaction, rather than failures of perception per se.

This reframing has important conceptual implications. Variability in perception, whether across individuals, developmental trajectories, or artificial agents, is not noise to be eliminated but an expected consequence of differences in embodiment, action strategies, and interaction histories. Autism, in this light, represents a distinct mode of perceptual organization that is internally coherent and adaptive within particular environmental contexts. This interpretation aligns with neurodiversity-informed perspectives and supports a shift away from deficit-based models toward accounts that emphasize process, variability, and lived experience.

The integration of embodied AI into this discussion further strengthens the framework by providing formal testbeds for examining perceptual self-organization. By manipulating parameters such as sensory precision, action variability, and temporal integration, embodied AI systems allow researchers to explore how continuous differences in interaction give rise to distinct perceptual profiles over time. These models do not reduce autism to an algorithmic impairment but instead offer a principled way to study how perception emerges from embodied dynamics across both biological and artificial systems.

### 9.1 Epistemological implications: perception as temporally structured interaction

Reframing perception as embodied, predictive, and temporally structured interaction carries substantive epistemological implications. Classical epistemologies of perception often assume that perceptual knowledge derives from the accurate internal representation of external states. Within such frameworks, perception is evaluated according to correspondence between internal content and objective reality. The interaction-centered account developed in this paper shifts explanatory priority from representational correspondence to relational regulation.

If perception emerges through lawful organism–environment coupling over time, then perceptual knowledge is not best understood as static depiction but as the stabilization of adaptive engagement. Knowing becomes a temporally extended achievement grounded in coordinated action, prediction, and feedback. Perceptual reliability, on this view, depends less on mirroring environmental structure and more on sustaining patterns of interaction that generate informative sensory consequences. Epistemic success is thus measured in terms of viable coordination rather than representational accuracy alone.

The temporal dimension is especially significant in this reconceptualization. Perception unfolds across multiple timescales, from moment-to-moment sensorimotor loops to developmental trajectories spanning years. Knowledge is therefore inherently dynamic, emerging from the accumulation and refinement of embodied expectations through repeated interaction. This perspective dissolves sharp distinctions between perception, action, and learning, treating them as phases of a continuous regulatory process.

Such an epistemology accommodates variability and diversity more readily than representational accounts. Differences in embodiment, temporal coordination, and predictive regulation will naturally yield distinct but coherent perceptual organizations. Rather than construing these differences as epistemic failures, an interaction-based framework interprets them as alternative modes of stabilizing organism–environment relations. This orientation aligns with ecological realism in acknowledging structured environmental constraints, while rejecting the necessity of detached internal reconstruction as the primary basis of perceptual knowledge.

By grounding epistemology in temporally extended interaction, the present framework provides a principled foundation for integrating biological perception, artificial intelligence, and neurodevelopmental diversity within a shared explanatory space. Perception becomes not the recovery of a pre-given world, but the ongoing enactment of meaningful engagement within structured environments.

### 9.2 Limitations and future directions

Several limitations of the present work should be acknowledged. First, the paper is theoretical in scope and does not present new empirical data. While the framework is grounded in a substantial body of recent empirical research, its core claims require further validation through experimental, developmental, and computational studies explicitly designed to test interaction-centered hypotheses. Second, autism spectrum disorder is highly heterogeneous, and no single framework can capture the full diversity of perceptual experiences observed across individuals. The embodied account advanced here is therefore not intended as a comprehensive theory of autism, but as a unifying lens that highlights foundational perceptual processes relevant to neurodevelopmental diversity.

A further limitation concerns the abstraction inherent in artificial intelligence models. Although embodied AI systems provide valuable formal tools, they necessarily simplify biological, social, and experiential complexity. Artificial agents cannot replicate lived perceptual experience, sociocultural context, or developmental history in full. As such, AI should be understood as a complementary methodology for theory refinement and hypothesis generation, rather than as a proxy for human cognition or development.

Future research should therefore pursue several converging directions. Longitudinal studies integrating fine-grained measures of movement, sensory responsivity, predictive regulation, and timing are needed to clarify how embodied interaction shapes perceptual development across typical and autistic populations. Experimental paradigms that allow active exploration and temporal coordination will be essential for testing embodied predictions. In parallel, embodied AI models should be used to formalize and explore how variations in sensorimotor coupling and temporal integration give rise to stable yet diverse perceptual organizations over time. Such work will benefit from interdisciplinary collaboration and from participatory approaches that incorporate the perspectives of autistic individuals themselves.

## 10 Conclusion

This paper has argued that perception is best understood as an embodied, predictive, and temporally coordinated process that emerges through self-organizing interaction with the environment. By integrating embodied cognition with contemporary AI-driven models of perception, the framework moves beyond representation-centric explanations toward a process-oriented account in which action, prediction, and timing jointly shape perceptual experience. This shift provides a coherent explanation for both the robustness of biological perception and the brittleness of disembodied artificial systems.

Extending this framework to autism spectrum disorder demonstrates its capacity to accommodate perceptual diversity without resorting to deficit-based interpretations. Autism emerges, within this account, as a difference in how perceptual systems self-organize through embodied interaction across development, rather than as a failure of perceptual mechanisms. Embodied AI models further enrich this perspective by offering formal tools for exploring how perceptual organization arises from interaction rather than abstraction.

More broadly, understanding perception as self-organizing interaction supports a reorientation of perceptual science, artificial intelligence, and neurodevelopmental theory toward interaction-centered explanations. By grounding perception in embodiment, prediction, and time, this framework provides a principled basis for studying sensory cognition across biological and artificial systems while respecting variability, development, and lived experience.
